# Supplementary material for: Genetic Diversity of Merozoite Surface Antigens in Global Babesia bovis Populations
Source: Genes (Basel). 2023 Oct 13;14(10):1936. doi: 10.3390/genes14101936 (PMC10606690; doi:10.3390/genes14101936)
Supplement: Supplementary file 1 [file genes-14-01936-s001.zip › genes-2593345-supplementary.pdf]

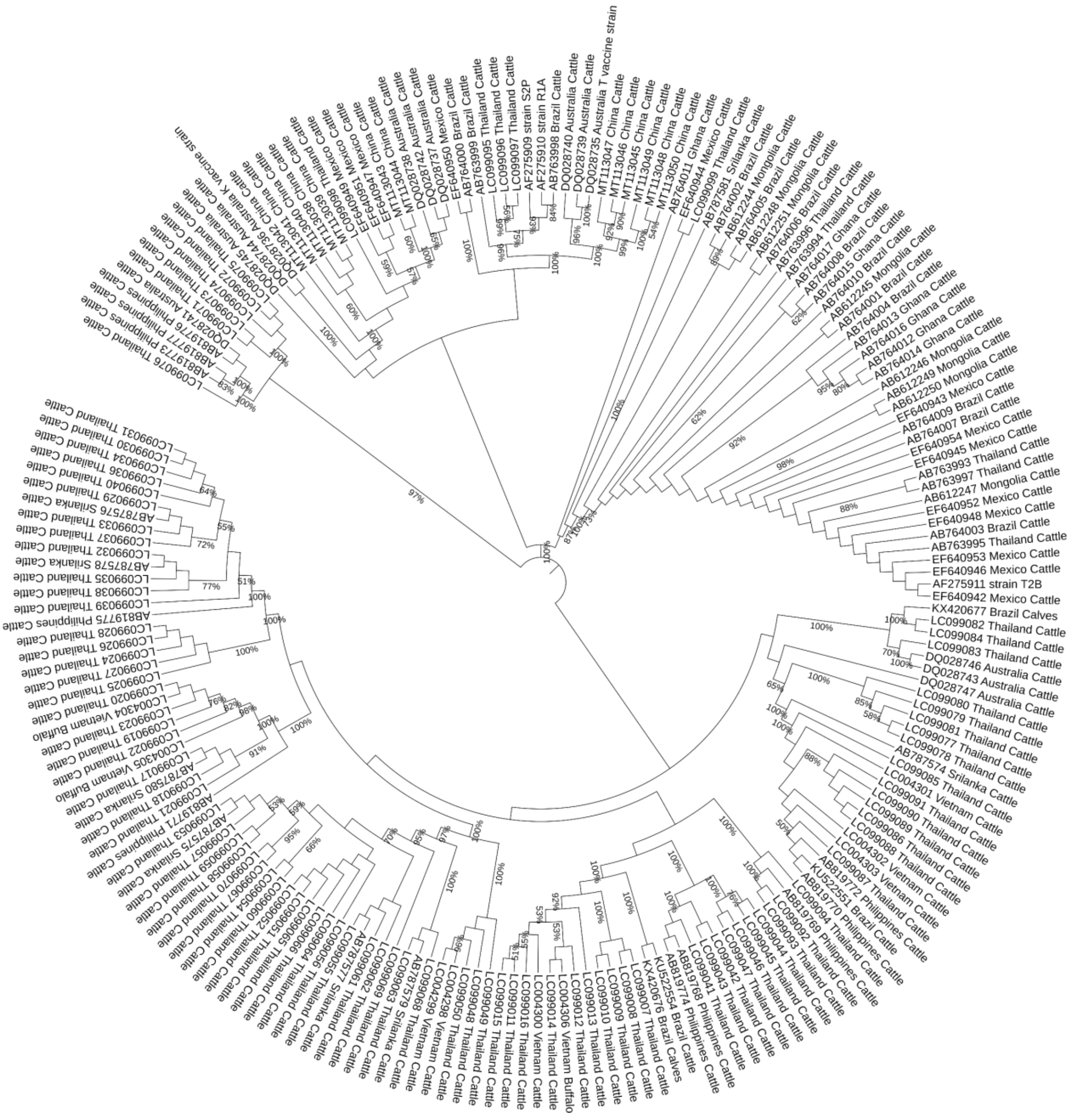

Figure S1. An unrooted phylogenetic tree of *B. bovis* MSA-1 gene constructed using the Maximum likelihood method based on Hasegawa-Kishino-Yano (HKY) Model. Numbers at the nodes represent the bootstrap values with more than 50% bootstrap support from 1000 replicates.

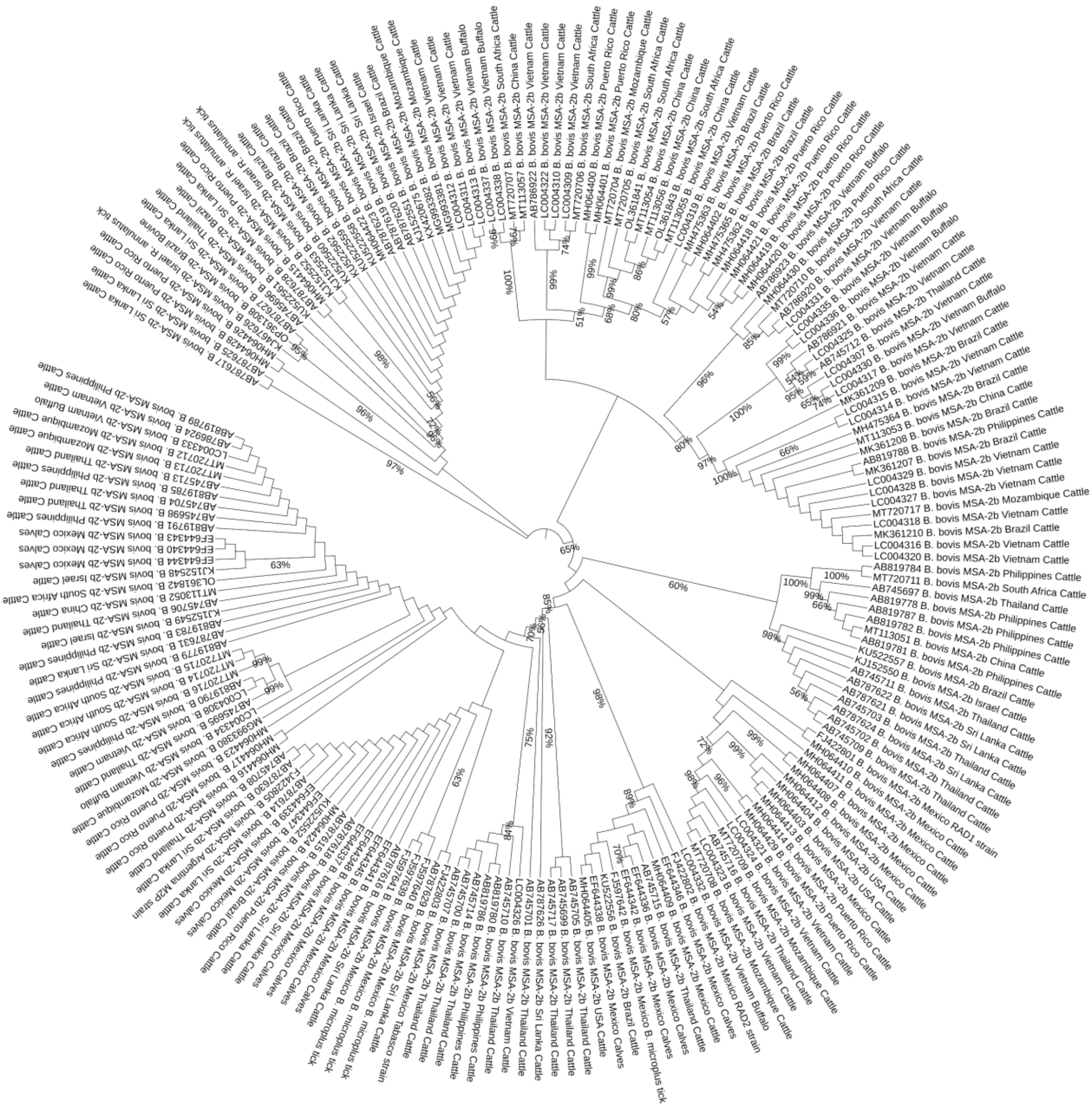

Figure S2. An unrooted phylogenetic tree of *B. bovis* MSA-2b gene fragment constructed using the Maximum likelihood method based on Hasegawa-Kishino-Yano (HKY) Model. Numbers at the nodes represent the bootstrap values with more than 50% bootstrap support from 1000 replicates.

Supplementary Table S1. Data describing the GenBank retrieved nucleotide sequences of *Babesia bovis* isolates that tested for various MSA fragments.

Country	Host	GenBank accession number	Reference
**MSA-1 isolates**			
Brazil	Cattle	KX420677	Matos et al., 2017
Brazil	Cattle	KX420676	Matos et al., 2017
Thailand	Cattle	LC099099	Tattiyapong et al., 2016
Thailand	Cattle	LC099098	Tattiyapong et al., 2016
Thailand	Cattle	LC099097	Tattiyapong et al., 2016
Thailand	Cattle	LC099096	Tattiyapong et al., 2016
Thailand	Cattle	LC099095	Tattiyapong et al., 2016
Thailand	Cattle	LC099094	Tattiyapong et al., 2016
Thailand	Cattle	LC099093	Tattiyapong et al., 2016
Thailand	Cattle	LC099092	Tattiyapong et al., 2016
Thailand	Cattle	LC099091	Tattiyapong et al., 2016
Thailand	Cattle	LC099090	Tattiyapong et al., 2016
Thailand	Cattle	LC099089	Tattiyapong et al., 2016
Thailand	Cattle	LC099088	Tattiyapong et al., 2016
Thailand	Cattle	LC099087	Tattiyapong et al., 2016
Thailand	Cattle	LC099086	Tattiyapong et al., 2016
Thailand	Cattle	LC099085	Tattiyapong et al., 2016
Thailand	Cattle	LC099084	Tattiyapong et al., 2016
Thailand	Cattle	LC099083	Tattiyapong et al., 2016
Thailand	Cattle	LC099082	Tattiyapong et al., 2016
Thailand	Cattle	LC099081	Tattiyapong et al., 2016
Thailand	Cattle	LC099080	Tattiyapong et al., 2016
Thailand	Cattle	LC099079	Tattiyapong et al., 2016
Thailand	Cattle	LC099078	Tattiyapong et al., 2016
Thailand	Cattle	LC099077	Tattiyapong et al., 2016
Thailand	Cattle	LC099076	Tattiyapong et al., 2016
Thailand	Cattle	LC099075	Tattiyapong et al., 2016
Thailand	Cattle	LC099074	Tattiyapong et al., 2016
Thailand	Cattle	LC099073	Tattiyapong et al., 2016
Thailand	Cattle	LC099072	Tattiyapong et al., 2016
Thailand	Cattle	LC099071	Tattiyapong et al., 2016
Thailand	Cattle	LC099070	Tattiyapong et al., 2016
Thailand	Cattle	LC099069	Tattiyapong et al., 2016
Thailand	Cattle	LC099068	Tattiyapong et al., 2016
Thailand	Cattle	LC099067	Tattiyapong et al., 2016
Thailand	Cattle	LC099066	Tattiyapong et al., 2016
Thailand	Cattle	LC099065	Tattiyapong et al., 2016
Thailand	Cattle	LC099064	Tattiyapong et al., 2016
Thailand	Cattle	LC099063	Tattiyapong et al., 2016
Thailand	Cattle	LC099062	Tattiyapong et al., 2016
Thailand	Cattle	LC099061	Tattiyapong et al., 2016
Thailand	Cattle	LC099060	Tattiyapong et al., 2016
Thailand	Cattle	LC099059	Tattiyapong et al., 2016

Thailand	Cattle	LC099058	Tattiyapong et al., 2016
Thailand	Cattle	LC099057	Tattiyapong et al., 2016
Thailand	Cattle	LC099056	Tattiyapong et al., 2016
Thailand	Cattle	LC099055	Tattiyapong et al., 2016
Thailand	Cattle	LC099054	Tattiyapong et al., 2016
Thailand	Cattle	LC099053	Tattiyapong et al., 2016
Thailand	Cattle	LC099052	Tattiyapong et al., 2016
Thailand	Cattle	LC099051	Tattiyapong et al., 2016
Thailand	Cattle	LC099050	Tattiyapong et al., 2016
Thailand	Cattle	LC099049	Tattiyapong et al., 2016
Thailand	Cattle	LC099048	Tattiyapong et al., 2016
Thailand	Cattle	LC099047	Tattiyapong et al., 2016
Thailand	Cattle	LC099046	Tattiyapong et al., 2016
Thailand	Cattle	LC099045	Tattiyapong et al., 2016
Thailand	Cattle	LC099044	Tattiyapong et al., 2016
Thailand	Cattle	LC099043	Tattiyapong et al., 2016
Thailand	Cattle	LC099042	Tattiyapong et al., 2016
Thailand	Cattle	LC099041	Tattiyapong et al., 2016
Thailand	Cattle	LC099040	Tattiyapong et al., 2016
Thailand	Cattle	LC099039	Tattiyapong et al., 2016
Thailand	Cattle	LC099038	Tattiyapong et al., 2016
Thailand	Cattle	LC099037	Tattiyapong et al., 2016
Thailand	Cattle	LC099036	Tattiyapong et al., 2016
Thailand	Cattle	LC099035	Tattiyapong et al., 2016
Thailand	Cattle	LC099034	Tattiyapong et al., 2016
Thailand	Cattle	LC099033	Tattiyapong et al., 2016
Thailand	Cattle	LC099032	Tattiyapong et al., 2016
Thailand	Cattle	LC099031	Tattiyapong et al., 2016
Thailand	Cattle	LC099030	Tattiyapong et al., 2016
Thailand	Cattle	LC099029	Tattiyapong et al., 2016
Thailand	Cattle	LC099028	Tattiyapong et al., 2016
Thailand	Cattle	LC099027	Tattiyapong et al., 2016
Thailand	Cattle	LC099026	Tattiyapong et al., 2016
Thailand	Cattle	LC099025	Tattiyapong et al., 2016
Thailand	Cattle	LC099024	Tattiyapong et al., 2016
Thailand	Cattle	LC099023	Tattiyapong et al., 2016
Thailand	Cattle	LC099022	Tattiyapong et al., 2016
Thailand	Cattle	LC099021	Tattiyapong et al., 2016
Thailand	Cattle	LC099020	Tattiyapong et al., 2016
Thailand	Cattle	LC099019	Tattiyapong et al., 2016
Thailand	Cattle	LC099018	Tattiyapong et al., 2016
Thailand	Cattle	LC099017	Tattiyapong et al., 2016
Thailand	Cattle	LC099016	Tattiyapong et al., 2016
Thailand	Cattle	LC099015	Tattiyapong et al., 2016
Thailand	Cattle	LC099014	Tattiyapong et al., 2016
Thailand	Cattle	LC099013	Tattiyapong et al., 2016
Thailand	Cattle	LC099012	Tattiyapong et al., 2016
Thailand	Cattle	LC099011	Tattiyapong et al., 2016
Thailand	Cattle	LC099010	Tattiyapong et al., 2016
Thailand	Cattle	LC099009	Tattiyapong et al., 2016

Thailand	Cattle	LC099008	Tattiyapong et al., 2016
Thailand	Cattle	LC099007	Tattiyapong et al., 2016
Ghana	Cattle	AB764017	Nagano et al., 2013
Ghana	Cattle	AB764016	Nagano et al., 2013
Ghana	Cattle	AB764015	Nagano et al., 2013
Ghana	Cattle	AB764014	Nagano et al., 2013
Ghana	Cattle	AB764013	Nagano et al., 2013
Ghana	Cattle	AB764012	Nagano et al., 2013
Ghana	Cattle	AB764011	Nagano et al., 2013
Brazil	Cattle	AB764010	Nagano et al., 2013
Brazil	Cattle	AB764009	Nagano et al., 2013
Brazil	Cattle	AB764008	Nagano et al., 2013
Brazil	Cattle	AB764007	Nagano et al., 2013
Brazil	Cattle	AB764006	Nagano et al., 2013
Brazil	Cattle	AB764005	Nagano et al., 2013
Brazil	Cattle	AB764004	Nagano et al., 2013
Brazil	Cattle	AB764003	Nagano et al., 2013
Brazil	Cattle	AB764002	Nagano et al., 2013
Brazil	Cattle	AB764001	Nagano et al., 2013
Brazil	Cattle	AB764000	Nagano et al., 2013
Brazil	Cattle	AB763999	Nagano et al., 2013
Brazil	Cattle	AB763998	Nagano et al., 2013
Thailand	Cattle	AB763997	Nagano et al., 2013
Thailand	Cattle	AB763996	Nagano et al., 2013
Thailand	Cattle	AB763995	Nagano et al., 2013
Thailand	Cattle	AB763994	Nagano et al., 2013
Thailand	Cattle	AB763993	Nagano et al., 2013
Mongolia	Cattle	AB612251	Altangerel et al., 2012
Mongolia	Cattle	AB612250	Altangerel et al., 2012
Mongolia	Cattle	AB612249	Altangerel et al., 2012
Mongolia	Cattle	AB612248	Altangerel et al., 2012
Mongolia	Cattle	AB612247	Altangerel et al., 2012
Mongolia	Cattle	AB612246	Altangerel et al., 2012
Mongolia	Cattle	AB612245	Altangerel et al., 2012
Mongolia	Cattle	AB612244	Altangerel et al., 2012
Mexico	Cattle	EF640954	Borgonio et al., 2008
Mexico	Cattle	EF640953	Borgonio et al., 2008
Mexico	Cattle	EF640952	Borgonio et al., 2008
Mexico	Cattle	EF640951	Borgonio et al., 2008
Mexico	Cattle	EF640950	Borgonio et al., 2008
Mexico	Cattle	EF640949	Borgonio et al., 2008
Mexico	Cattle	EF640948	Borgonio et al., 2008
Mexico	Cattle	EF640947	Borgonio et al., 2008
Mexico	Cattle	EF640946	Borgonio et al., 2008
Mexico	Cattle	EF640945	Borgonio et al., 2008
Mexico	Cattle	EF640944	Borgonio et al., 2008
Vietnam	Buffalo	LC004306	Yokoyama et al., 2015
Vietnam	Buffalo	LC004305	Yokoyama et al., 2015
Vietnam	Buffalo	LC004304	Yokoyama et al., 2015
Vietnam	Cattle	LC004303	Yokoyama et al., 2015

Vietnam	Cattle	LC004302	Yokoyama et al., 2015
Vietnam	Cattle	LC004301	Yokoyama et al., 2015
Vietnam	Cattle	LC004300	Yokoyama et al., 2015
Vietnam	Cattle	LC004299	Yokoyama et al., 2015
Vietnam	Cattle	LC004298	Yokoyama et al., 2015
Sri Lanka	Cattle	AB787581	Sivakumar et al., 2013
Sri Lanka	Cattle	AB787580	Sivakumar et al., 2013
Sri Lanka	Cattle	AB787579	Sivakumar et al., 2013
Sri Lanka	Cattle	AB787578	Sivakumar et al., 2013
Sri Lanka	Cattle	AB787577	Sivakumar et al., 2013
Sri Lanka	Cattle	AB787576	Sivakumar et al., 2013
Sri Lanka	Cattle	AB787575	Sivakumar et al., 2013
Sri Lanka	Cattle	AB787574	Sivakumar et al., 2013
Philippines	Cattle	AB819777	Tattiyapong et al., 2014
Philippines	Cattle	AB819776	Tattiyapong et al., 2014
Philippines	Cattle	AB819775	Tattiyapong et al., 2014
Philippines	Cattle	AB819774	Tattiyapong et al., 2014
Philippines	Cattle	AB819773	Tattiyapong et al., 2014
Philippines	Cattle	AB819772	Tattiyapong et al., 2014
Philippines	Cattle	AB819771	Tattiyapong et al., 2014
Philippines	Cattle	AB819770	Tattiyapong et al., 2014
Philippines	Cattle	AB819769	Tattiyapong et al., 2014
Philippines	Cattle	AB819768	Tattiyapong et al., 2014
China	Cattle	MT113050	Wang et al., 2020
China	Cattle	MT113049	Wang et al., 2020
China	Cattle	MT113048	Wang et al., 2020
China	Cattle	MT113047	Wang et al., 2020
China	Cattle	MT113046	Wang et al., 2020
China	Cattle	MT113045	Wang et al., 2020
China	Cattle	MT113044	Wang et al., 2020
China	Cattle	MT113043	Wang et al., 2020
China	Cattle	MT113042	Wang et al., 2020
China	Cattle	MT113041	Wang et al., 2020
China	Cattle	MT113040	Wang et al., 2020
China	Cattle	MT113039	Wang et al., 2020
China	Cattle	MT113038	Wang et al., 2020
Brazil	Cattle	KU522554	Unpublished
Brazil	Cattle	KU522551	Unpublished
Mexico	Cattle	EF640943	Borgonio et al., 2008
Mexico	Cattle	EF640942	Borgonio et al., 2008
Australia	T vaccine strain	DQ028735	LeRoith et al., 2005
Australia	K vaccine strain	DQ028736	LeRoith et al., 2005
Australia	Cattle	DQ028737	LeRoith et al., 2005
Australia	Cattle	DQ028738	LeRoith et al., 2005
Australia	Cattle	DQ028739	LeRoith et al., 2005
Australia	Cattle	DQ028740	LeRoith et al., 2005
Australia	Cattle	DQ028741	LeRoith et al., 2005
Australia	Cattle	DQ028742	LeRoith et al., 2005
Australia	Cattle	DQ028743	LeRoith et al., 2005
Australia	Cattle	DQ028744	LeRoith et al., 2005

Australia	Cattle	DQ028745	LeRoith et al., 2005
Australia	Cattle	DQ028746	LeRoith et al., 2005
Australia	Cattle	DQ028747	LeRoith et al., 2005
Argentina	strain S2P	AF275909	Suarez et al., 2000
Argentina	strain R1A	AF275910	Suarez et al., 2000
USA	strain T2B	AF275911	Suarez et al., 2000
**MSA-2a1 isolates**			
Israel	Cattle calves	KJ156373	Molad et al., 2014
Israel	Cattle calves	KJ156372	Molad et al., 2014
Israel	Cattle calves	KJ156371	Molad et al., 2014
Israel	Cattle calves	KJ144252	Molad et al., 2015
Israel	Cattle	KJ156370	Molad et al., 2014
Israel	R. annulatus ticks	KJ152555	Molad et al., 2014
Israel	R. annulatus ticks	KJ152554	Molad et al., 2014
Sri Lanka	Cattle	AB787610	Sivakumar et al., 2013
Sri Lanka	Cattle	AB787609	Sivakumar et al., 2013
Sri Lanka	Cattle	AB787608	Sivakumar et al., 2013
Sri Lanka	Cattle	AB787607	Sivakumar et al., 2013
Sri Lanka	Cattle	AB787606	Sivakumar et al., 2013
Sri Lanka	Cattle	AB787605	Sivakumar et al., 2013
Sri Lanka	Cattle	AB787604	Sivakumar et al., 2013
Sri Lanka	Cattle	AB787603	Sivakumar et al., 2013
Sri Lanka	Cattle	AB787602	Sivakumar et al., 2013
Sri Lanka	Cattle	AB787601	Sivakumar et al., 2013
Sri Lanka	Cattle	AB787600	Sivakumar et al., 2013
Mexico	Calves	FJ422809	Dominguez et al., 2010
Argentina	Calves	FJ422808	Dominguez et al., 2010
Argentina	Calves	FJ422807	Dominguez et al., 2010
Argentina	Calves	FJ422806	Dominguez et al., 2010
Mexico	strain Mo7	AY052538	Florin-Christensen et al., 2002
Argentina	strain R1A	AY052539	Florin-Christensen et al., 2002
**MSA-2b isolates**			
Puerto Rico	Cattle	MH064430	Unpublished
Puerto Rico	Cattle	MH064429	Unpublished
Puerto Rico	Cattle	MH064428	Unpublished
Puerto Rico	Cattle	MH064426	Unpublished
Puerto Rico	Cattle	MH064424	Unpublished
Puerto Rico	Cattle	MH064423	Unpublished
Puerto Rico	Cattle	MH064422	Unpublished
Puerto Rico	Cattle	MH064421	Unpublished
Puerto Rico	Cattle	MH064420	Unpublished
Puerto Rico	Cattle	MH064419	Unpublished
Puerto Rico	Cattle	MH064418	Unpublished
Puerto Rico	Cattle	MH064417	Unpublished
Puerto Rico	Cattle	MH064416	Unpublished
Puerto Rico	Cattle	MH064415	Unpublished

Puerto Rico	Cattle	MH064414	Unpublished
Brazil	Cattle	KX420675	Matos et al., 2017
Mexico	B. microplus tick	FJ597642	Genis et al., 2009
Mexico	B. microplus tick	FJ597641	Genis et al., 2009
Mexico	B. microplus tick	FJ597640	Genis et al., 2009
Mexico	B. microplus tick	FJ597639	Genis et al., 2009
Thailand	Cattle	AB745717	Simking et al., 2013
Thailand	Cattle	AB745716	Simking et al., 2013
Thailand	Cattle	AB745715	Simking et al., 2013
Thailand	Cattle	AB745714	Simking et al., 2013
Thailand	Cattle	AB745713	Simking et al., 2013
Thailand	Cattle	AB745712	Simking et al., 2013
Thailand	Cattle	AB745711	Simking et al., 2013
Thailand	Cattle	AB745710	Simking et al., 2013
Thailand	Cattle	AB745709	Simking et al., 2013
Thailand	Cattle	AB745708	Simking et al., 2013
Thailand	Cattle	AB745707	Simking et al., 2013
Thailand	Cattle	AB745706	Simking et al., 2013
Thailand	Cattle	AB745705	Simking et al., 2013
Thailand	Cattle	AB745704	Simking et al., 2013
Thailand	Cattle	AB745703	Simking et al., 2013
Thailand	Cattle	AB745702	Simking et al., 2013
Thailand	Cattle	AB745701	Simking et al., 2013
Thailand	Cattle	AB745700	Simking et al., 2013
Thailand	Cattle	AB745699	Simking et al., 2013
Thailand	Cattle	AB745698	Simking et al., 2013
Thailand	Cattle	AB745697	Simking et al., 2013
Thailand	Cattle	AB745696	Simking et al., 2013
Thailand	Cattle	AB745695	Simking et al., 2013
South Africa	Cattle	OL361843	Byaruhanga et al., 2023
South Africa	Cattle	OL361842	Byaruhanga et al., 2023
South Africa	Cattle	OL361841	Byaruhanga et al., 2023
Mozambique	Cattle	MT720717	Byaruhanga et al., 2023
South Africa	Cattle	MT720716	Byaruhanga et al., 2023
South Africa	Cattle	MT720715	Byaruhanga et al., 2023
South Africa	Cattle	MT720714	Byaruhanga et al., 2023
Mozambique	Cattle	MT720713	Byaruhanga et al., 2023
Mozambique	Cattle	MT720712	Byaruhanga et al., 2023
South Africa	Cattle	MT720711	Byaruhanga et al., 2023
South Africa	Cattle	MT720710	Byaruhanga et al., 2023
Mozambique	Cattle	MT720709	Byaruhanga et al., 2023
Mozambique	Cattle	MT720708	Byaruhanga et al., 2023
South Africa	Cattle	MT720707	Byaruhanga et al., 2023
South Africa	Cattle	MT720706	Byaruhanga et al., 2023
South Africa	Cattle	MT720705	Byaruhanga et al., 2023
Mozambique	Cattle	MT720704	Byaruhanga et al., 2023

Brazil	Cattle	MK361210	Mendes et al., 2019
Brazil	Cattle	MK361209	Mendes et al., 2019
Brazil	Cattle	MK361208	Mendes et al., 2019
Brazil	Cattle	MK361207	Mendes et al., 2019
Mexico	Cattle	MH064413	Unpublished
Mexico	Cattle	MH064412	Unpublished
Mexico	Cattle	MH064411	Unpublished
Mexico	Cattle	MH064410	Unpublished
Mexico	Cattle	MH064409	Unpublished
Mexico	Cattle	MH064408	Unpublished
Mexico	Cattle	MH064407	Unpublished
USA	Cattle	MH064405	Unpublished
USA	Cattle	MH064404	Unpublished
USA	Cattle	MH064403	Unpublished
Puerto Rico	Cattle	MH064402	Unpublished
Puerto Rico	Cattle	MH064401	Unpublished
Puerto Rico	Cattle	MH064400	Unpublished
Brazil	Cattle	MH475365	Mendes et al., 2019
Brazil	Cattle	MH475364	Mendes et al., 2019
Brazil	Cattle	MH475363	Mendes et al., 2019
Brazil	Cattle	MH475362	Mendes et al., 2019
Mozambique	Cattle	MG993382	Unpublished
Mozambique	Cattle	MG993381	Unpublished
Mozambique	Cattle	MG993380	Unpublished
Mexico	Calves	EF644348	Genis et al., 2008
Mexico	Calves	EF644347	Genis et al., 2008
Mexico	Calves	EF644346	Genis et al., 2008
Mexico	Calves	EF644345	Genis et al., 2008
Mexico	Calves	EF644344	Genis et al., 2008
Mexico	Calves	EF644343	Genis et al., 2008
Mexico	Calves	EF644342	Genis et al., 2008
Mexico	Calves	EF644341	Genis et al., 2008
Mexico	Calves	EF644340	Genis et al., 2008
Mexico	Calves	EF644339	Genis et al., 2008
Mexico	Calves	EF644338	Genis et al., 2008
Mexico	Calves	EF644337	Genis et al., 2008
Mexico	Calves	EF644336	Genis et al., 2008
Vietnam	Buffalo	LC004338	Yokoyama et al., 2015
Vietnam	Buffalo	LC004337	Yokoyama et al., 2015
Vietnam	Buffalo	LC004336	Yokoyama et al., 2015
Vietnam	Buffalo	LC004335	Yokoyama et al., 2015
Vietnam	Buffalo	LC004334	Yokoyama et al., 2015
Vietnam	Buffalo	LC004333	Yokoyama et al., 2015
Vietnam	Buffalo	LC004332	Yokoyama et al., 2015
Vietnam	Buffalo	LC004331	Yokoyama et al., 2015
Vietnam	Buffalo	LC004330	Yokoyama et al., 2015
Vietnam	Cattle	LC004329	Yokoyama et al., 2015
Vietnam	Cattle	LC004328	Yokoyama et al., 2015
Vietnam	Cattle	LC004327	Yokoyama et al., 2015
Vietnam	Cattle	LC004326	Yokoyama et al., 2015

Vietnam	Cattle	LC004325	Yokoyama et al., 2015
Vietnam	Cattle	LC004324	Yokoyama et al., 2015
Vietnam	Cattle	LC004323	Yokoyama et al., 2015
Vietnam	Cattle	LC004322	Yokoyama et al., 2015
Vietnam	Cattle	LC004321	Yokoyama et al., 2015
Vietnam	Cattle	LC004320	Yokoyama et al., 2015
Vietnam	Cattle	LC004319	Yokoyama et al., 2015
Vietnam	Cattle	LC004318	Yokoyama et al., 2015
Vietnam	Cattle	LC004317	Yokoyama et al., 2015
Vietnam	Cattle	LC004316	Yokoyama et al., 2015
Vietnam	Cattle	LC004315	Yokoyama et al., 2015
Vietnam	Cattle	LC004314	Yokoyama et al., 2015
Vietnam	Cattle	LC004313	Yokoyama et al., 2015
Vietnam	Cattle	LC004312	Yokoyama et al., 2015
Vietnam	Cattle	LC004311	Yokoyama et al., 2015
Vietnam	Cattle	LC004310	Yokoyama et al., 2015
Vietnam	Cattle	LC004309	Yokoyama et al., 2015
Vietnam	Cattle	LC004308	Yokoyama et al., 2015
Vietnam	Cattle	LC004307	Yokoyama et al., 2015
Israel	R. annulatus tick	KJ152553	Molad et al., 2014
Israel	R. annulatus tick	KJ152552	Molad et al., 2014
Israel	Cattle	KJ152551	Molad et al., 2014
Israel	Cattle	KJ152550	Molad et al., 2014
Israel	Cattle	KJ152549	Molad et al., 2014
Israel	Cattle	KJ152548	Molad et al., 2014
Sri Lanka	Cattle	AB787631	Sivakumar et al., 2013
Sri Lanka	Cattle	AB787630	Sivakumar et al., 2013
Sri Lanka	Cattle	AB787629	Sivakumar et al., 2013
Sri Lanka	Cattle	AB787628	Sivakumar et al., 2013
Sri Lanka	Cattle	AB787627	Sivakumar et al., 2013
Sri Lanka	Cattle	AB787626	Sivakumar et al., 2013
Sri Lanka	Cattle	AB787625	Sivakumar et al., 2013
Sri Lanka	Cattle	AB787624	Sivakumar et al., 2013
Sri Lanka	Cattle	AB787623	Sivakumar et al., 2013
Sri Lanka	Cattle	AB787622	Sivakumar et al., 2013
Sri Lanka	Cattle	AB787621	Sivakumar et al., 2013
Sri Lanka	Cattle	AB787620	Sivakumar et al., 2013
Sri Lanka	Cattle	AB787619	Sivakumar et al., 2013
Sri Lanka	Cattle	AB787618	Sivakumar et al., 2013
Sri Lanka	Cattle	AB787617	Sivakumar et al., 2013
Sri Lanka	Cattle	AB787616	Sivakumar et al., 2013
Sri Lanka	Cattle	AB787615	Sivakumar et al., 2013
Sri Lanka	Cattle	AB787614	Sivakumar et al., 2013
Philippines	Cattle	AB819791	Tattiyapong et al., 2014
Philippines	Cattle	AB819790	Tattiyapong et al., 2014
Philippines	Cattle	AB819789	Tattiyapong et al., 2014
Philippines	Cattle	AB819788	Tattiyapong et al., 2014
Philippines	Cattle	AB819787	Tattiyapong et al., 2014

Philippines	Cattle	AB819786	Tattiyapong et al., 2014
Philippines	Cattle	AB819785	Tattiyapong et al., 2014
Philippines	Cattle	AB819784	Tattiyapong et al., 2014
Philippines	Cattle	AB819783	Tattiyapong et al., 2014
Philippines	Cattle	AB819782	Tattiyapong et al., 2014
Philippines	Cattle	AB819781	Tattiyapong et al., 2014
Philippines	Cattle	AB819780	Tattiyapong et al., 2014
Philippines	Cattle	AB819779	Tattiyapong et al., 2014
Philippines	Cattle	AB819778	Tattiyapong et al., 2014
Brazil	Bovine	OP361308	Unpublished
Argentina	Strain M2P	FJ422805	Dominguez et al., 2010
Mexico	Tabasco strain	FJ422803	Dominguez et al., 2010
Mexico	Strain RAD2	FJ422802	Dominguez et al., 2010
Mexico	Strain RAD1	FJ422801	Dominguez et al., 2010
Vietnam	Cattle	AB786924	Sivakumar et al., 2013
Vietnam	Buffalo	AB786923	Sivakumar et al., 2013
Vietnam	Cattle	AB786922	Sivakumar et al., 2013
Vietnam	Cattle	AB786921	Sivakumar et al., 2013
Vietnam	Cattle	AB786920	Sivakumar et al., 2013
China	Cattle	MT113057	Wang et al., 2020
China	Cattle	MT113056	Wang et al., 2020
China	Cattle	MT113055	Wang et al., 2020
China	Cattle	MT113054	Wang et al., 2020
China	Cattle	MT113053	Wang et al., 2020
China	Cattle	MT113052	Wang et al., 2020
China	Cattle	MT113051	Wang et al., 2020
Brazil	Cattle	KU522562	Unpublished
Brazil	Cattle	KU522561	Unpublished
Brazil	Cattle	KU522560	Unpublished
Brazil	Cattle	KU522559	Unpublished
Brazil	Cattle	KU522558	Unpublished
Brazil	Cattle	KU522557	Unpublished
Brazil	Cattle	KU522556	Unpublished
Brazil	Cattle	KU522552	Unpublished
Israel	R. annulatus tick	KJ467626	Molad et al., 2014
**MSA-2c isolates**			
Brazil	Cattle	KX420674	Matos et al., 2017
Brazil	Cattle	KX420673	Matos et al., 2017
Brazil	Cattle	KX420672	Matos et al., 2017
Israel	R. annulatus ticks	KJ144257	Molad et al., 2014
Israel	Calf	KJ144256	Molad et al., 2014
Israel	Cattle	KJ144255	Molad et al., 2014
Israel	Calf	KJ144254	Molad et al., 2014
Israel	R. annulatus ticks	KJ144253	Molad et al., 2014
Mongolia	Cattle	AB612260	Altangerel et al., 2012
Mongolia	Cattle	AB612259	Altangerel et al., 2012
Mongolia	Cattle	AB612258	Altangerel et al., 2012

Mongolia	Cattle	AB612257	Altangerel et al., 2012
Mongolia	Cattle	AB612256	Altangerel et al., 2012
Mongolia	Cattle	AB612255	Altangerel et al., 2012
Mongolia	Cattle	AB612254	Altangerel et al., 2012
Mongolia	Cattle	AB612253	Altangerel et al., 2012
Mongolia	Cattle	AB612252	Altangerel et al., 2012
Mexico	Cattle	EF640967	Borgonio et al., 2008
Mexico	Cattle	EF640966	Borgonio et al., 2008
Mexico	Cattle	EF640965	Borgonio et al., 2008
Mexico	Cattle	EF640964	Borgonio et al., 2008
Mexico	Cattle	EF640963	Borgonio et al., 2008
Mexico	Cattle	EF640962	Borgonio et al., 2008
Mexico	Cattle	EF640961	Borgonio et al., 2008
Mexico	Cattle	EF640960	Borgonio et al., 2008
Mexico	Cattle	EF640959	Borgonio et al., 2008
Mexico	Cattle	EF640958	Borgonio et al., 2008
Mexico	Cattle	EF640957	Borgonio et al., 2008
Mexico	Cattle	EF640956	Borgonio et al., 2008
Mexico	Cattle	EF640955	Borgonio et al., 2008
Brazil	Cattle	MK305092	Mendes et al., 2019
Brazil	Cattle	MK305091	Mendes et al., 2019
Brazil	Cattle	MK305090	Mendes et al., 2019
Brazil	Cattle	MH751591	Mendes et al., 2019
Brazil	Cattle	MH751590	Mendes et al., 2019
Brazil	Cattle	MH751589	Mendes et al., 2019
Brazil	Cattle	MH751588	Mendes et al., 2019
Brazil	Cattle	MH751587	Mendes et al., 2019
Brazil	Calves	KX463635	Matos et al., 2020
Brazil	Calves	KX463634	Matos et al., 2020
Brazil	Calves	KX463633	Matos et al., 2020
Brazil	Calves	KX463632	Matos et al., 2020
Turkey	Cattle	GU647154	Duzlu et al., 2011
Turkey	Cattle	GU647153	Duzlu et al., 2011
Turkey	Cattle	GU647152	Duzlu et al., 2011
Turkey	Cattle	GU647151	Duzlu et al., 2011
Turkey	Cattle	GU647150	Duzlu et al., 2011
Turkey	Cattle	GU647149	Duzlu et al., 2011
Turkey	Cattle	GU647148	Duzlu et al., 2011
Turkey	Cattle	GU647147	Duzlu et al., 2011
Turkey	Cattle	GU357634	Duzlu et al., 2011
Turkey	Cattle	GU004533	Duzlu et al., 2011
Turkey	Cattle	KC515392	Duzlu et al., 2015
Turkey	Cattle	KC515391	Duzlu et al., 2015
Turkey	Cattle	KC515390	Duzlu et al., 2015
Turkey	Cattle	KC515389	Duzlu et al., 2015
Vietnam	Buffalo	LC004373	Yokoyama et al., 2015
Vietnam	Buffalo	LC004372	Yokoyama et al., 2015
Vietnam	Buffalo	LC004371	Yokoyama et al., 2015
Vietnam	Buffalo	LC004370	Yokoyama et al., 2015
Vietnam	Buffalo	LC004369	Yokoyama et al., 2015

Vietnam	Buffalo	LC004368	Yokoyama et al., 2015
Vietnam	Buffalo	LC004367	Yokoyama et al., 2015
Vietnam	Buffalo	LC004366	Yokoyama et al., 2015
Vietnam	Buffalo	LC004365	Yokoyama et al., 2015
Vietnam	Buffalo	LC004364	Yokoyama et al., 2015
Vietnam	Buffalo	LC004363	Yokoyama et al., 2015
Vietnam	Cattle	LC004362	Yokoyama et al., 2015
Vietnam	Cattle	LC004361	Yokoyama et al., 2015
Vietnam	Cattle	LC004360	Yokoyama et al., 2015
Vietnam	Cattle	LC004359	Yokoyama et al., 2015
Vietnam	Cattle	LC004358	Yokoyama et al., 2015
Vietnam	Cattle	LC004357	Yokoyama et al., 2015
Vietnam	Cattle	LC004356	Yokoyama et al., 2015
Vietnam	Cattle	LC004355	Yokoyama et al., 2015
Vietnam	Cattle	LC004354	Yokoyama et al., 2015
Vietnam	Cattle	LC004353	Yokoyama et al., 2015
Vietnam	Cattle	LC004352	Yokoyama et al., 2015
Vietnam	Cattle	LC004351	Yokoyama et al., 2015
Vietnam	Cattle	LC004350	Yokoyama et al., 2015
Vietnam	Cattle	LC004349	Yokoyama et al., 2015
Vietnam	Cattle	LC004348	Yokoyama et al., 2015
Vietnam	Cattle	LC004347	Yokoyama et al., 2015
Vietnam	Cattle	LC004346	Yokoyama et al., 2015
Vietnam	Cattle	LC004345	Yokoyama et al., 2015
Vietnam	Cattle	LC004344	Yokoyama et al., 2015
Vietnam	Cattle	LC004343	Yokoyama et al., 2015
Vietnam	Cattle	LC004342	Yokoyama et al., 2015
Vietnam	Cattle	LC004341	Yokoyama et al., 2015
Vietnam	Cattle	LC004340	Yokoyama et al., 2015
Vietnam	Cattle	LC004339	Yokoyama et al., 2015
Sri Lanka	Cattle	AB787599	Sivakumar et al., 2013
Sri Lanka	Cattle	AB787598	Sivakumar et al., 2013
Sri Lanka	Cattle	AB787597	Sivakumar et al., 2013
Sri Lanka	Cattle	AB787596	Sivakumar et al., 2013
Sri Lanka	Cattle	AB787595	Sivakumar et al., 2013
Sri Lanka	Cattle	AB787594	Sivakumar et al., 2013
Sri Lanka	Cattle	AB787593	Sivakumar et al., 2013
Sri Lanka	Cattle	AB787592	Sivakumar et al., 2013
Sri Lanka	Cattle	AB787591	Sivakumar et al., 2013
Sri Lanka	Cattle	AB787590	Sivakumar et al., 2013
Sri Lanka	Cattle	AB787589	Sivakumar et al., 2013
Sri Lanka	Cattle	AB787588	Sivakumar et al., 2013
Sri Lanka	Cattle	AB787587	Sivakumar et al., 2013
Sri Lanka	Cattle	AB787586	Sivakumar et al., 2013
Sri Lanka	Cattle	AB787585	Sivakumar et al., 2013
Sri Lanka	Cattle	AB787584	Sivakumar et al., 2013
Sri Lanka	Cattle	AB787583	Sivakumar et al., 2013
Sri Lanka	Cattle	AB787582	Sivakumar et al., 2013
Philippines	Cattle	AB819805	Tattiyapong et al., 2014
Philippines	Cattle	AB819804	Tattiyapong et al., 2014

Philippines	Cattle	AB819803	Tattiyapong et al., 2014
Philippines	Cattle	AB819802	Tattiyapong et al., 2014
Philippines	Cattle	AB819801	Tattiyapong et al., 2014
Philippines	Cattle	AB819800	Tattiyapong et al., 2014
Philippines	Cattle	AB819799	Tattiyapong et al., 2014
Philippines	Cattle	AB819798	Tattiyapong et al., 2014
Philippines	Cattle	AB819797	Tattiyapong et al., 2014
Philippines	Cattle	AB819796	Tattiyapong et al., 2014
Philippines	Cattle	AB819795	Tattiyapong et al., 2014
Philippines	Cattle	AB819794	Tattiyapong et al., 2014
Philippines	Cattle	AB819793	Tattiyapong et al., 2014
Philippines	Cattle	AB819792	Tattiyapong et al., 2014
Turkey	Bovine	HM117270	Yavuz et al., 2011
Brazil	Bovine	OP361309	Unpublished
Argentina	Strain M2P	FJ411372	Dominguez el al., 2010
Mexico	Strain Veracruz	FJ422800	Dominguez el al., 2010
Mexico	Strain RAD	FJ422799	Dominguez el al., 2010
Mexico	Strain Tabasco	FJ422798	Dominguez el al., 2010
Mexico	Strain Pullman	FJ422797	Dominguez el al., 2010
Argentina	Strain S2P	FJ422796	Dominguez el al., 2010
Argentina	Strain M1A	FJ422795	Dominguez el al., 2010
Argentina	Strain M3P	FJ422794	Dominguez el al., 2010
China	Cattle	MT113065	Wang et al., 2020
China	Cattle	MT113064	Wang et al., 2020
China	Cattle	MT113063	Wang et al., 2020
China	Cattle	MT113062	Wang et al., 2020
China	Cattle	MT113061	Wang et al., 2020
China	Cattle	MT113060	Wang et al., 2020
China	Cattle	MT113059	Wang et al., 2020
China	Cattle	MT113058	Wang et al., 2020
Brazil	Cattle	KU522567	Matos et al., 2017
Brazil	Cattle	KU522566	Matos et al., 2017
Brazil	Cattle	KU522565	Matos et al., 2017
Brazil	Cattle	KU522564	Matos et al., 2017
Brazil	Cattle	KU522563	Matos et al., 2017
Israel	R. annulatus ticks	KJ467627	Molad et al., 2014
Argentina	Strain R1A	AY052542	Florin-Christensen et al., 2002

Supplementary Table S2. Genetic indices among different populations of *Babesia bovis* calculated after amplification of the MSA-1 gene. Numbers of the tested isolates from each population are given in parenthesis.

Population 1	Population 2	Fst	Gst	Kxy	Nm	Dxy	Da
**Continent-related indices**							
South America (17)	Africa (7)	0.09163	0.04131	129.21010	5.80	0.22668	0.02077
South America	North America (13)	0.05487	0.02362	153.28050	10.34	0.26611	0.01460
South America	Australia (13)	0.14179	0.01702	229.32130	14.44	0.40877	0.05796
South America	Asia (146)	0.24756	0.01218	245.74580	20.27	0.45008	0.11142
Africa	North America	0.06665	0.05045	117.36260	4.71	0.16369	0.01091
Africa	Australia	0.37539	0.05045	218.87910	4.71	0.39438	0.14805
Africa	Asia	0.51057	0.03669	263.01170	6.56	0.48171	0.24595
North America	Australia	0.23367	0.04348	216.50890	5.50	0.36266	0.08474
North America	Asia	0.42885	0.02374	267.90200	10.28	0.45562	0.19539
Australia	Asia	0.16913	0.01819	251.61850	13.50	0.43684	0.07388
**Country-related indices**							
Sri Lanka (8)	Brazil (17)	0.32390	0.00751	266.38240	33.05	0.46981	0.15217
Sri Lanka	Mongolia (8)	0.64779	0.00901	324.98440	27.50	0.47512	0.30778
Sri Lanka	Thailand (98)	-0.02594	0.02144	178.34820	11.41	0.29725	-0.00771
Sri Lanka	Ghana (7)	0.56113	0.03697	315.00000	6.51	0.50971	0.28601
Sri Lanka	Mexico (13)	0.48915	0.01512	310.12500	16.28	0.48533	0.23740
Sri Lanka	Australia (13)	0.25901	0.01201	267.29810	20.57	0.45692	0.11834
Sri Lanka	China (13)	0.49019	0.02465	332.64420	9.89	0.51097	0.25048
Sri Lanka	Philippines (10)	0.08173	0.01748	206.41250	14.05	0.32302	0.02640
Sri Lanka	Vietnam (9)	0.05428	0.00718	200.22220	34.59	0.30615	0.01662
Brazil	Mongolia	0.19140	0.00890	115.84560	27.85	0.20008	0.03829
Brazil	Thailand	0.33134	0.01194	266.99400	20.68	0.47848	0.15854
Brazil	Ghana	0.09163	0.04131	129.21010	5.80	0.22668	0.02077
Brazil	Mexico	0.05487	0.02362	153.28050	10.34	0.26611	0.01460
Brazil	Australia	0.14179	0.01702	229.32130	14.44	0.40877	0.05796
Brazil	China	0.30973	0.04477	209.10860	5.33	0.36880	0.11423

Brazil	Philippines	0.40626	0.01458	287.07650	16.89	0.50631	0.20569
Brazil	Vietnam	0.40972	0.01195	282.70590	20.68	0.49081	0.20110
Mongolia	Thailand	0.67668	0.02956	298.16960	8.21	0.49695	0.33628
Mongolia	Ghana	0.03313	0.04663	49.23214	5.11	0.06461	0.00214
Mongolia	Mexico	0.20117	0.02377	106.54810	10.27	0.13252	0.02666
Mongolia	Australia	0.48748	0.02929	229.93270	8.29	0.38131	0.18588
Mongolia	China	0.65981	0.02060	288.77880	11.89	0.35131	0.23180
Mongolia	Philippines	0.72685	0.02666	346.12500	9.13	0.54167	0.39371
Mongolia	Vietnam	0.72977	0.01627	351.90280	15.12	0.52366	0.38216
Thailand	Ghana	0.59038	0.03785	294.05540	6.35	0.52698	0.31112
Thailand	Mexico	0.51095	0.02675	299.26220	9.10	0.49877	0.25485
Thailand	Australia	0.24493	0.01849	260.56280	13.27	0.45237	0.11080
Thailand	China	0.51109	0.02901	295.29280	8.37	0.50220	0.25667
Thailand	Philippines	0.04655	0.02200	182.13060	11.11	0.30355	0.01413
Thailand	Vietnam	0.04267	175.65310	183.49140	10.44	0.29276	0.01249
Ghana	Mexico	0.06665	0.05045	117.36260	4.71	0.16369	0.01091
Ghana	Australia	0.37539	0.05045	218.87910	4.71	0.39438	0.14805
Ghana	China	0.52850	0.06041	261.02200	3.89	0.35369	0.18692
Ghana	Philippines	0.64967	0.05496	330.87140	4.30	0.56851	0.36934
Ghana	Vietnam	0.65684	0.04447	330.46030	5.37	0.55633	0.36542
Mexico	Australia	0.23367	0.04348	216.50890	5.50	0.36266	0.08474
Mexico	China	0.31346	0.02295	238.91720	10.64	0.30397	0.09528
Mexico	Philippines	0.57520	0.05128	327.49230	4.63	0.53512	0.30780
Mexico	Vietnam	0.58129	0.04110	327.29910	5.83	0.52201	0.30344
Australia	China	0.10431	0.02717	194.44970	8.95	0.32087	0.03347
Australia	Philippines	0.29284	0.02721	270.62310	8.94	0.45791	0.13409
Australia	Vietnam	0.32054	0.01781	282.75210	13.79	0.46891	0.15030
China	Philippines	0.55684	0.04078	337.82310	5.88	0.53623	0.29859
China	Vietnam	0.56048	0.03093	342.68370	7.83	0.53129	0.29778
Philippines	Vietnam	0.06719	0.02428	193.63330	10.05	0.29608	0.01989

Fst, Wright's F-statistics for pairwise genetic distance; Gst, genetic differentiation index based on the frequency of haplotypes; Nm, gene flow value; Kxy, average proportion of nucleotide differences between populations; Dxy, average number of nucleotide substitutions per site between populations; Da, number of net nucleotide substitutions per site between populations.

Supplementary Table S3. Genetic indices among different populations of *Babesia bovis* calculated after amplification of the MSA-2a1 fragment of the MSA-2 gene. Numbers of the tested isolates from each population are given in parenthesis.

Population 1	Population 2	Fst	Gst	Kxy	Nm	Dxy	Da
**Country-related indices**							
Sri Lanka (11)	Mexico (2)	0.25088	0.06821	79.86364	3.41	0.11046	0.02771
Sri Lanka	Argentina (4)	0.19120	0.02168	58.00000	11.28	0.08022	0.01534
Sri Lanka	Israel (7)	0.15370	0.00720	98.03896	34.46	0.13560	0.02084
Mexico	Argentina	0.60273	0.02041	116.00000	12.00	0.16044	0.09670
Mexico	Israel	-0.09838	0.04726	74.78571	5.04	0.10344	-0.01018
Argentina	Israel	0.40396	0.00725	116.14290	34.22	0.16064	0.06489

Fst, Wright's F-statistics for pairwise genetic distance; Gst, genetic differentiation index based on the frequency of haplotypes; Nm, gene flow value; Kxy, average proportion of nucleotide differences between populations; Dxy, average number of nucleotide substitutions per site between populations; Da, number of net nucleotide substitutions per site between populations.

Supplementary Table S4. Genetic indices among different populations of *Babesia bovis* calculated after amplification of the MSA-2b region of the MSA-2 gene. Numbers of the tested isolates from each population are given in parenthesis.

Population 1	Population 2	Fst	Gst	Kxy	Nm	Dxy	Da
**Host-related indices**							
Cattle (176)	Water buffalo (10)	0.04398	0.02210	76.85114	11.06	0.19261	0.00847
Cattle	Ticks (7)	0.00088	0.03317	69.10958	7.29	0.17364	0.00015
Water buffalo	Ticks	0.10223	0.01901	81.68571	12.90	0.20070	0.02052
**Continents-related indices**							
North America (48)	Asia (106)	0.04874	0.00753	75.06387	32.93	0.18443	0.00899
North America	South America (19)	0.15886	0.01782	79.84430	13.78	0.20061	0.03187
North America	Africa (20)	0.03293	0.01574	73.74895	15.64	0.18120	0.00597
Asia	South America	0.06888	0.01391	74.81132	17.72	0.18750	0.01291
Asia	Africa	0.01275	0.00869	74.79151	28.51	0.18331	0.00234
South America	Africa	0.05990	0.01730	74.16842	14.20	0.18589	0.01113
**Country-related indices**							
Puerto Rico (18)	China (7)	-0.01757	0.02436	76.53175	10.01	0.18758	-0.00330
Puerto Rico	Mexico (27)	0.25877	0.01279	83.50617	19.30	0.20517	0.05309
Puerto Rico	USA (3)	0.06224	0.08837	79.38889	2.58	0.19458	0.01211
Puerto Rico	Thailand (23)	0.18954	0.01261	82.30676	19.58	0.20173	0.03824
Puerto Rico	Sri Lanka (18)	0.11316	0.01078	79.14198	22.93	0.19398	0.02195
Puerto Rico	Vietnam (37)	0.09372	0.01303	85.16817	18.94	0.20875	0.01956
Puerto Rico	Israel (7)	0.07176	0.02120	78.58730	11.54	0.19262	0.01382
Puerto Rico	South Africa (11)	0.00896	0.02343	75.31313	10.42	0.18459	0.00165
Puerto Rico	Philippines (14)	0.19642	0.03210	83.52381	7.54	0.20472	0.04021
Puerto Rico	Brazil (18)	0.07105	0.01923	76.24383	12.75	0.19109	0.01358
Puerto Rico	Mozambique (9)	-0.00794	0.02378	77.98148	10.26	0.19113	-0.00152
China	Mexico	0.28225	0.03008	84.91534	8.06	0.20864	0.05889
China	USA	0.07499	0.10020	79.38095	2.25	0.19456	0.01459
China	Thailand	0.19674	0.01988	81.77640	12.33	0.20043	0.03943
China	Sri Lanka	0.15223	0.03132	81.58730	7.73	0.19997	0.03044
China	Vietnam	0.03774	0.02840	79.15444	8.55	0.19401	0.00732
China	Israel	0.12107	0.02439	81.83673	10.00	0.20058	0.02428
China	South Africa	-0.06076	0.00701	69.40260	35.42	0.17010	-0.01034

China	Philippines	0.16038	0.00679	78.72449	36.56	0.19295	0.03095
China	Brazil	0.04343	0.03706	73.50000	6.50	0.18421	0.00800
China	Mozambique	-0.00074	0.01845	77.52381	13.30	0.19001	-0.00014
Mexico	USA	0.11868	0.08134	65.04939	2.82	0.15983	0.01897
Mexico	Thailand	0.06182	0.01392	52.95813	17.71	0.13012	0.00804
Mexico	Sri Lanka	0.16624	0.00575	63.74691	43.21	0.15663	0.02604
Mexico	Vietnam	0.29164	0.01997	85.07207	1.17	0.20902	0.06096
Mexico	Israel	0.24448	0.02494	74.02116	1.50	0.18187	0.04446
Mexico	South Africa	0.21283	0.02979	73.17171	8.14	0.17978	0.03826
Mexico	Philippines	0.13556	0.03538	57.92064	6.82	0.14231	0.01929
Mexico	Brazil	0.36377	0.02749	85.29836	8.84	0.21432	0.07796
Mexico	Mozambique	0.12976	0.03308	70.74486	7.31	0.17382	0.02255
USA	Thailand	0.16080	0.08681	74.18841	2.55	0.18183	0.02924
USA	Sri Lanka	0.14130	0.09325	76.55556	2.43	0.18764	0.02651
USA	Vietnam	0.12115	0.08450	82.76576	2.71	0.20286	0.02458
USA	Israel	0.13862	0.10020	79.52381	2.25	0.19491	0.02702
USA	South Africa	0.09942	0.10160	77.93939	2.21	0.19103	0.01899
USA	Philippines	0.20599	0.10349	78.92857	2.17	0.19345	0.03985
USA	Brazil	0.17589	0.09734	81.24074	2.32	0.20361	0.03581
USA	Mozambique	-0.03096	0.10654	71.92593	2.10	0.17629	-0.00546
Thailand	Sri Lanka	0.05714	0.01612	61.51208	15.26	0.15076	0.00861
Thailand	Vietnam	0.21060	0.01067	82.33842	23.18	0.20181	0.04250
Thailand	Israel	0.14730	0.02645	71.25466	9.20	0.17464	0.02572
Thailand	South Africa	0.13502	0.02641	72.19763	9.22	0.17696	0.02389
Thailand	Philippines	0.00767	0.01293	55.35404	19.08	0.13567	0.00104
Thailand	Brazil	0.27970	0.02761	81.94927	8.80	0.20539	0.05745
Thailand	Mozambique	0.06672	0.01431	71.15942	17.22	0.17441	0.01164
Sri Lanka	Vietnam	0.16525	0.01776	82.03304	13.83	0.20106	0.03322
Sri Lanka	Israel	-0.04224	0.02168	61.63492	11.28	0.15107	-0.00638
Sri Lanka	South Africa	0.11558	0.03403	74.54546	7.10	0.18271	0.02112
Sri Lanka	Philippines	0.09114	0.04079	64.26588	5.88	0.15751	0.01436
Sri Lanka	Brazil	0.14016	0.01089	72.69136	22.70	0.18218	0.02553
Sri Lanka	Mozambique	-0.00057	0.02042	69.85185	11.99	0.17121	-0.00010
Vietnam	Israel	0.13749	0.02738	82.59460	8.88	0.20244	0.02783
Vietnam	South Africa	0.08175	0.02451	79.42261	9.95	0.19466	0.01591
Vietnam	Philippines	0.19669	0.02232	81.42471	10.95	0.19957	0.03925
Vietnam	Brazil	0.05171	0.01567	73.06757	15.70	0.18313	0.00947
Vietnam	Mozambique	0.03151	0.01251	79.39339	19.73	0.19459	0.00613
Israel	South Africa	0.10686	0.03233	76.90909	7.48	0.18850	0.02014
Israel	Philippines	0.16299	0.03977	73.08163	6.04	0.17912	0.02919
Israel	Brazil	0.03821	0.02076	67.75397	11.79	0.16981	0.00649
Israel	Mozambique	-0.02374	0.01845	70.96825	13.30	0.17394	-0.00413
South Africa	Philippines	0.11697	0.04482	71.18831	5.33	0.17448	0.02041
South Africa	Brazil	0.11650	0.04077	75.95454	5.88	0.19036	0.02218

South Africa	Mozambique	-0.00492	0.03564	73.97980	6.77	0.18132	-0.00089
Philippines	Brazil	0.25931	0.04592	80.87302	5.19	0.20269	0.05256
Philippines	Mozambique	0.07871	0.02041	72.53175	12.00	0.17777	0.01399
Brazil	Mozambique	0.03400	0.01236	73.24691	19.97	0.18358	0.00624

Fst, Wright's F-statistics for pairwise genetic distance; Gst, genetic differentiation index based on the frequency of haplotypes; Nm, gene flow value; Kxy, average proportion of nucleotide differences between populations; Dxy, average number of nucleotide substitutions per site between populations; Da, number of net nucleotide substitutions per site between populations.

Supplementary Table S5. Genetic indices among different populations of *Babesia bovis* calculated after amplification of the MSA-2c region of the MSA-2 gene. Numbers of the tested isolates from each population are given in parenthesis.

Population 1	Population 2	Fst	Gst	Kxy	Nm	Dxy	Da
**Host-related indices**							
Cattle (134)	Water buffalo (11)	0.18502	0.01889	53.59227	12.98	0.08728	0.01615
Cattle	Ticks (3)	-0.06390	0.07228	101.16670	3.21	0.16477	-0.01053
Water buffalo	Ticks	0.11556	0.03637	106.33330	6.62	0.17206	0.01988
**Continent-related indices**							
Europe (15)	Asia (91)	0.25834	0.01032	61.63663	23.98	0.09990	0.02581
Europe	North America (17)	0.29436	0.00565	33.98431	44.01	0.05499	0.01619
Europe	South America (26)	0.20110	0.00763	37.25128	32.50	0.06057	0.01218
Asia	North America	0.09377	0.01101	54.48933	22.45	0.08831	0.00828
Asia	South America	0.06965	0.00835	58.94886	29.71	0.09601	0.00669
North America	South America	0.00660	0.00141	33.49321	176.75	0.05446	0.00036
**Country-related indices**							
Vietnam (35)	Philippines (14)	0.08368	0.00471	72.14490	52.87	0.11674	0.00977
Vietnam	China (8)	0.35890	0.02170	130.22140	11.27	0.21071	0.07563
Vietnam	Mongolia (9)	0.52379	0.01366	36.88254	18.05	0.05968	0.03126
Vietnam	Sri Lanka (18)	0.23404	0.00741	60.64444	33.47	0.09829	0.02300
Vietnam	Israel (6)	0.16555	0.02505	70.89524	9.73	0.11472	0.01899
Vietnam	Turkey (15)	0.48379	0.00591	47.94286	42.07	0.07758	0.03753
Vietnam	Mexico (17)	0.29188	0.00896	40.12773	27.65	0.06493	0.01895
Vietnam	Brazil (21)	0.24742	0.01079	47.12517	22.91	0.07663	0.01896
Vietnam	Argentina (5)	0.47421	0.04255	43.53714	5.62	0.07045	0.03341
Philippines	China	0.08459	0.01447	131.53570	17.03	0.21284	0.01800
Philippines	Mongolia	0.27988	0.00477	75.66666	52.17	0.12244	0.03427
Philippines	Sri Lanka	0.07823	0.00416	90.45238	59.84	0.14660	0.01147
Philippines	Israel	-0.06724	0.01390	71.61111	17.74	0.11606	-0.00780
Philippines	Turkey	0.25468	0.00279	82.74762	89.30	0.13390	0.03410

Philippines	Mexico	0.15814	0.00647	77.61345	38.38	0.12559	0.01986
Philippines	Brazil	0.10625	0.01031	80.38776	23.99	0.13071	0.01389
Philippines	Argentina	0.24063	0.03616	78.77143	6.66	0.12746	0.03067
China	Mongolia	0.45476	0.00917	131.80560	27.02	0.21328	0.09699
China	Sri Lanka	0.24353	0.01841	133.18750	13.33	0.21586	0.05257
China	Israel	0.15329	0.01031	134.00000	24.00	0.21683	0.03324
China	Turkey	0.41607	0.01248	135.37500	19.78	0.21905	0.09114
China	Mexico	0.37662	0.01852	132.69120	13.25	0.21471	0.08087
China	Brazil	0.31652	0.02244	130.08330	10.89	0.21152	0.06695
China	Argentina	0.41432	0.03709	131.80000	6.49	0.21327	0.08836
Mongolia	Sri Lanka	0.15414	0.00896	41.17901	27.64	0.06674	0.01029
Mongolia	Israel	0.11540	0.00277	53.74074	90.00	0.08696	0.01004
Mongolia	Turkey	0.62046	0.00277	34.59259	90.00	0.05598	0.03473
Mongolia	Mexico	0.18472	0.00901	20.60131	27.51	0.03334	0.00616
Mongolia	Brazil	0.11508	0.01334	26.94709	18.50	0.04382	0.00504
Mongolia	Argentina	0.61011	0.02928	28.91111	8.29	0.04678	0.02854
Sri Lanka	Israel	-0.06724	0.01390	71.61111	17.74	0.11606	-0.00780
Sri Lanka	Turkey	0.23073	0.00514	54.61852	48.39	0.08852	0.02042
Sri Lanka	Mexico	0.02060	0.00726	46.64379	34.16	0.07560	0.00156
Sri Lanka	Brazil	0.00227	0.01085	52.44709	22.79	0.08542	0.00019
Sri Lanka	Argentina	0.18815	0.03989	49.46667	6.02	0.08017	0.01508
Israel	Turkey	0.06116	0.01060	58.28889	23.33	0.09432	0.00577
Israel	Mexico	-0.00232	0.01693	58.25490	14.52	0.09426	-0.00022
Israel	Brazil	-0.04944	0.02196	61.88095	11.14	0.10062	-0.00497
Israel	Argentina	0.02580	0.02604	54.26667	9.35	0.08781	0.00227
Turkey	Mexico	0.29436	0.00565	33.98431	44.01	0.05499	0.01619
Turkey	Brazil	0.25178	0.00897	41.47301	27.61	0.06744	0.01698
Turkey	Argentina	0.05445	0.03465	19.52000	6.96	0.03159	0.00172
Mexico	Brazil	0.00566	0.00013	34.73949	1858.72	0.05649	0.00032
Mexico	Argentina	0.22197	0.03992	28.43529	6.01	0.04601	0.01021
Brazil	Argentina	0.19579	0.04320	36.27619	5.54	0.05899	0.01155

Fst, Wright’s F-statistics for pairwise genetic distance; Gst, genetic differentiation index based on the frequency of haplotypes; Nm, gene flow value; Kxy, average proportion of nucleotide differences between populations; Dxy, average number of nucleotide substitutions per site between populations; Da, number of net nucleotide substitutions per site between populations.

Supplementary Table S6. Intra-population genetic diversity of *B. bovis* MSA genes isolates based on their country of origin

Fragment/Country	n	S	Eta	*p* (%)	K	π	H	Hd	C
**MSA1**									
Ghana	7	288	292	0.0 – 37.4	85.23810	0.11186	5	0.857	0.295
Vietnam	9	382	463	0.0 – 37.3	194.05556	0.27882	8	0.972	0.157
Philippines	10	392	548	0.0 – 38.7	186.86667	0.28313	7	0.933	0.135
Sri Lanka	8	493	671	0.1 – 55.4	219.46429	0.32085	8	1.000	0.141
Thailand	98	509	929	0.0 – 57.3	186.59268	0.31099	54	0.981	0.096
China	13	390	432	0.0 – 40.4	195.89744	0.23074	11	0.962	0.211
Mongolia	8	41	41	0.0 – 2.2	10.96429	0.01265	7	0.964	0.393
Mexico	13	434	552	0.0 – 37.7	161.01282	0.20026	10	0.949	0.170
Brazil	17	469	718	0.0 – 60.9	181.13235	0.31284	16	0.993	0.150
Australia	13	476	711	0.0 – 58.9	233.21795	0.38295	10	0.962	0.129
**MSA-2b**									
South Africa	11	155	181	0.0 – 29.7	67.03636	0.16552	8	0.927	0.617
Mozambique	9	165	191	0.0 – 29.3	76.30556	0.18841	6	0.917	0.593
Vietnam	37	203	263	0.0 – 30.3	71.46246	0.17645	20	0.940	0.499
Thailand	23	180	219	0.0 – 29.0	53.49802	0.13209	16	0.953	0.556
Sri Lanka	18	152	168	0.0 – 29.7	63.39216	0.15652	12	0.922	0.625
Philippines	14	131	148	0.0 – 28.4	53.17582	0.13130	7	0.824	0.677
China	7	154	184	0.0 – 29.3	71.09524	0.17554	6	0.952	0.620
Israel	7	143	188	0.0 – 31.4	68.333	0.16872	6	0.952	0.647
Mexico	27	131	141	0.0 – 30.9	45.32479	0.11191	19	0.957	0.675
Puerto Rico	18	185	213	0.0 – 34.4	69.19608	0.17085	15	0.974	0.543
Brazil	18	184	217	0.0 – 29.5	65.90196	0.16517	12	0.915	0.534
**MSA-2c**									
Vietnam	35	99	107	0.0 – 9.1	29.18319	0.04722	30	0.990	0.840
Sri Lanka	18	262	277	0.0 – 48.9	63.71895	0.10327	15	0.980	0.576
Philippines	14	285	336	0.0 – 46.8	103.03297	0.16672	13	0.989	0.539

China-Mongolia	17	264	296	0.0 – 47.6	99.72059	0.16136	16	0.993	0.573
Turkey	15	110	118	0.2 – 9.9%	20.31429	0.03287	15	1.000	0.822
Mexico	17	104	111	0.0 – 11.1	27.64706	0.04474	15	0.978	0.832
Brazil	21	270	298	0.0 – 46.0	41.74762	0.06788	16	0.967	0.558
Vietnam	35	99	107	0.0 – 9.1	29.18319	0.04722	30	0.990	0.840

n, number of sequences tested; L, base pair length of the aligned sequences; S, number of segregating (polymorphic/variable) sites; Eta, total number of mutations; *p*, pairwise distance; π, nucleotide diversity; K, average number of pairwise nucleotide differences; H, number of haplotypes; Hd, haplotype diversity; C, sequence conservation.
